# Supplementary material for: Cost-Effectiveness Analysis of Hp and New Gastric Cancer Screening Scoring System for Screening and Prevention of Gastric Cancer
Source: Curr Oncol. 2023 Jan 13;30(1):1132–45. doi: 10.3390/curroncol30010086 (PMC9857951; doi:10.3390/curroncol30010086)
Supplement: Supplementary file 1 [file curroncol-30-00086-s001.zip › curroncol-2066228-supplementary.pdf]

Table S1. The parameters of Markov model.

Parameter		Baseline value	range	distribution	reference
basic probability					
Hp infection rate		0.5	0.4-0.6	triangle	[8,25,26]
14C screening sensitivity		0.96	0.95-0.96	β	[27]
14C screening specificity		0.93	0.91-0.94	β	[27]
Success rate of Hp eradication treatment		0.81	0.73-0.88	β	[28–30]
Incidence of GC in Hp positive population		0.0028	0.0018–0.0056	β	[30–34]
Incidence of GC after treatment		0.0017	0.0007–0.0036	β	[30–34]
Incidence of GC in Hp negative population		0.0001	0.00006-0.0007	β	[35]
Screening population group					
40-49	low risk	0.9499			
	moderate risk	0.0501			[11,36–38]
	high risk	0			
50-59	low risk	0.5556			
	moderate risk	0.4259			
	high risk	0.0185			
60-69	low risk	0.4454			
	moderate risk	0.4898			
	high risk	0.0648			
≥70	low risk	0.0286			
	moderate risk	0.5571			
	high risk	0.4143			
ESD resection rate		0.92	0.75-1.0	β	[39,40]
Prevalence					
	AG	0.25	0.08-0.8	β	[41,42]
	IM	0.35	0.14-0.48	β	[43–45]
	dysplasia	0.06	0.05-0.09	β	[45]
	GC	0.0003	0.0003-0.00037	β	[8,46]
low risk	AG	0.0928			[36–38,47,48]
	IM	0.0899			
	dysplasia	0.0683			
	GC	0.0017			
moderate risk	AG	0.1669			
	IM	0.1273			
	dysplasia	0.0901			
	GC	0.0087			
high risk	AG	0.3149			
	IM	0.2545			
	dysplasia	0.1699			
	GC	0.035			
GC stage at diagnosis	Stage I	II	III	IV	
Screening population	0.85	0.04	0.08	0.03	[15,49,50]
no screening population	0.07	0.17	0.33	0.43	
Transition probability					
Hp infected gastritis- AG		0.018	0.005-0.05	β	[42,51]
Hp uninfected gastritis-AG		0.005	0.001-0.05	β	
AG -IM		0.009	0.001-0.20	β	
AG- gastritis		0.0086	0.0005-0.0099	β	

IM- dysplasia		0.007	0.001-0.09	β	
dysplasia-GC		0.0026	0.0019-0.0035	β	[52]
Stage I-II		0.0241			[53,54]
Stage II-III		0.0468			
Stage III-IV		0.271			
Population mortality		0.00714			National Bureau of statistics data 2020
5-year survival rate	Stage I	0.933	0.903–0.963	β	
	Stage II	0.700	0.672–0.728	β	
	Stage III	0.412	0.388–0.436	β	
	Stage IV	0.152	0.135–0.169	β	
Cost (yuan / person / year)					
	14C	180	90-360	triangle	Charging standard of Taiyuan third class hospital
	NGCS	500	250-1000	triangle	
	Hp treatment	400	200-800	triangle	
	endoscopy	300	150-600	triangle	
	Pathology	117	58.5-234	triangle	
	Atrophy/ IM	5000	2500-10000	triangle	Expert advice
	ESD	15000	7500-30000	triangle	
Stage I	first year	87000	43500-174000	triangle	Expert advice , [15]
	subsequent year	12000	6000-24000	triangle	
Stage II	first year	163000	81500-326000	triangle	
	subsequent year	28000	14000-56000	triangle	
Stage III	first year	159000	79500-318000	triangle	
	subsequent year	18000	9000-36000	triangle	
Stage IV	first year	230000	115000-460000	triangle	
	subsequent year	44000	22000-88000	triangle	
utility					
	Hp infection	0.89	0.8–1.0	triangle	[55]
	gastritis/AG/IM/ dysplasia	0.98	0.9–1.0	β	[42]
	Stage I	0.88	0.6–1.0	β	[42,50,56]
	Stage II	0.86	0.62–0.99	β	
	Stage III	0.77	0.58–0.95	β	
	Stage IV	0.68	0.51–0.84	β	
	clinical diagnosis GC	0.49	0.17-0.79	β	[21]
discount rate		0.03	0-0.06		
Population life expectancy (years)		77.3	-	-	National Bureau of statistics data 2019
Per capita GDP		80976			National Bureau of statistics data 2020

Notes: AG, atrophic gatritis; IM, intestinal metaplasia; GC, Gastric cancer; Hp, helicobacter pylori; NGCS, new gastric cancer screening scoring system; ESD, endoscopic submucosal dissection; GDP, gross domestic product.
